# Innovative Perspective: Gadolinium-Free Magnetic Resonance Imaging in Long-Term Follow-Up after Kidney Transplantation

**DOI:** 10.3389/fphys.2017.00296

**Published:** 2017-05-16

**Authors:** Mick J. M. van Eijs, Arjan D. van Zuilen, Anneloes de Boer, Martijn Froeling, Tri Q. Nguyen, Jaap A. Joles, Tim Leiner, Marianne C. Verhaar

**Affiliations:** ^1^Department of Nephrology and Hypertension, University Medical Center UtrechtUtrecht, Netherlands; ^2^Department of Radiology, University Medical Center UtrechtUtrecht, Netherlands; ^3^Department of Pathology, University Medical Center UtrechtUtrecht, Netherlands

**Keywords:** magnetic resonance imaging, functional MRI, kidney transplantation follow-up, chronic allograft nephropathy, protocol kidney biopsy, chronic hypoxia theory

## Abstract

Since the mid-1980s magnetic resonance imaging (MRI) has been investigated as a non- or minimally invasive tool to probe kidney allograft function. Despite this long-standing interest, MRI still plays a subordinate role in daily practice of transplantation nephrology. With the introduction of new functional MRI techniques, administration of exogenous gadolinium-based contrast agents has often become unnecessary and true non-invasive assessment of allograft function has become possible. This raises the question why application of MRI in the follow-up of kidney transplantation remains restricted, despite promising results. Current literature on kidney allograft MRI is mainly focused on assessment of (sub) acute kidney injury after transplantation. The aim of this review is to survey whether MRI can provide valuable diagnostic information beyond 1 year after kidney transplantation from a mechanistic point of view. The driving force behind chronic allograft nephropathy is believed to be chronic hypoxia. Based on this, techniques that visualize kidney perfusion and oxygenation, scarring, and parenchymal inflammation deserve special interest. We propose that functional MRI mechanistically provides tools for diagnostic work-up in long-term follow-up of kidney allografts.

## Introduction

A rise in serum creatinine concentration (SCr) is often the first sign of kidney allograft dysfunction. It is only observed if considerable damage has occurred in the allograft, from which it follows that initial damage develops subclinically (Pascual et al., 2012). Various processes contribute to damage accumulating in kidney allografts over time, e.g., calcineurin inhibitor nephrotoxicity, chronic rejection, aging, and recurrent infections (Pascual et al., 2002). Early detection of allograft damage, preferably even before the actual onset of allograft fibrosis, is desirable, since it may improve long term allograft survival (Pascual et al., 2012). Currently the best method to monitor the course of such damage would be sequential protocol biopsies.

Protocol biopsy facilitates early diagnosis of *de novo* and recurrent kidney disease (Morozumi et al., 2014) and therefore one or sometimes multiple protocol biopsies several months to a year after kidney transplantation are performed in several programs. Specific indications for protocol biopsies have been proposed (Racusen, 2006). Although pathology findings currently are leading for therapy initiation or alteration, benefits of protocol biopsies for the early detection of subclinical allograft damage are seriously questioned due to considerable burden (Tanabe, 2014). Especially in the absence of any clinical signs indicating allograft dysfunction, kidney biopsy comes with risks and drawbacks, including a logistic burden for nephrology departments, discomfort in patients, risk of allograft bleeding, and, albeit to a lesser extent, infectious complications (Corapi et al., 2012; Chung et al., 2014; Morgan et al., 2016). Finally, there is a risk of sampling error which may confound adequate assessment of biopsy samples, (Madaio, 1990) since allograft fibrosis develops from focal lesions (Cosio et al., 2008). In practice this strategy is therefore not universally applied on a regular basis. A non-invasive alternative test such as MRI would be a more convenient solution. However, centers that perform protocol biopsies are provided with the ideal opportunity of conducting longitudinal imaging studies that could also involve parallel assessment of kidney imaging and histological morphology. Such parallel assessment could possibly also contribute to improvement in evaluation of acute kidney injury (AKI) and chronic kidney disease (CKD) in native kidneys.

MRI has been suggested as a promising technique in clinical nephrology, but thus far has not been successfully implemented as a specific method of choice in follow-up of kidney transplant recipients (Michaely et al., 2007; Chandarana and Lee, 2009; Zhang et al., 2014a). Whereas previous reviews on renal imaging discussed applications of MRI in the entire domain of nephrology, (Ebrahimi et al., 2014; Grenier et al., 2016) this review will specifically address disease processes underlying kidney allograft damage, focusing on different aspects that can be assessed with MRI techniques. A brief description of the technical background of these techniques will be provided. Our main aim is to evaluate—from a mechanistic point of view—whether MRI has the potential to provide valuable diagnostic information in long-term follow-up of kidney transplant recipients.

## Conventional vs. functional MRI for the assessment of subclinical kidney allograft damage

### Conventional MRI techniques

A distinction must be made between functional MRI techniques on the one hand, and conventional, non-functional, or anatomic MRI techniques on the other. Functional MRI refers to techniques that aim to measure or visualize physiological variables in the kidney, whereas non-functional MRI renders images of kidney anatomy. Conventional imaging of the kidney (through T_1_, T_2_ and proton density weighted imaging) (Currie et al., 2013) can identify morphological abnormalities, for instance with magnetic resonance urography and angiography, and a sensitive, though non-specific loss of corticomedullary differentiation with increased T_1_ relaxation times can be observed in kidneys with impaired function (Huang et al., 2011). Since T_1_ imaging cannot identify the cause of kidney function impairment given its lack of specificity, this technique is probably of little value in evaluation of allograft function in transplantation patients.

### Functional MRI techniques

In addition to assessment of kidney morphology investigators have often tried to assess single kidney renal function with dynamic contrast-enhanced MRI (DCE-MRI) (Zeng et al., 2015) but its advantage in kidney transplantation patients above eGFR is debatable, given that in this case total GFR depends almost entirely on allograft GFR. DCE-MRI depicts the passage of an intravenously injected gadolinium-based contrast agent (GBCA) through the vascular system and kidney parenchyma. DCE-MRI has been used for calculation of GFR (Eikefjord et al., 2015; Zeng et al., 2015) and to discriminate between acutely rejected and non-rejected kidney allografts (Khalifa et al., 2013). Although this technique is very promising, concerns about the long-term side effects of GBCAs by both clinicians as well as patients have stymied the adoption of DCE-MRI. It is also of note that detection of kidney pathology by assessment of a suspected decrease in GFR is of rather restricted use, since reduced GFR is in fact the final result of the entire chain of events in kidney disease.

Several other (patho)physiological processes in the kidney potentially relevant for kidney allograft follow-up have been visualized with non-contrast enhanced (functional) MRI to date (Table 1). The chronic hypoxia theory, first introduced by Fine et al. (1998) postulates that progressive development of fibrosis in chronic kidney disease is related to kidney allograft hypoxia (Fine and Norman, 2008). Indeed it was demonstrated in experimental diabetes that hypoxia had occurred at an early stage (dos Santos et al., 2007) before any histologic damage could be observed (Manotham et al., 2004). This has also been observed in experimental kidney transplantation (Papazova et al., 2015). Moreover, hypoxia-inducible factor-1alpha has been immunohistochemically detected in kidney allografts with (sub)clinical rejection at 3 months after transplantation and beyond (Rosenberger et al., 2007). There is, however, an unmet need for longitudinal functional imaging studies, both in experimental and clinical settings. These studies would contribute most to a better understanding of the role of hypoxia in the course of chronic allograft nephropathy (CAN).

**Table 1:** **Currently available techniques for kidney allograft imaging**.

**(Patho)physiological process**	**Imaging technique**
Oxygenation	Blood oxygen level dependent (BOLD) imaging
Water diffusion and tubular flow	Diffusion weighted imaging (DWI)/Diffusion tensor imaging (DTI)
(Arterial) blood supply	Arterial spin labeling (ASL)
Scarring	T_1_ in the rotating frame (T_1ρ_)
	Magnetic resonance elastography (MRE)
	Diffusion weighted imaging (DWI)/Diffusion tensor imaging (DTI)
Inflammation	Ultrasmall superparamagnetic particles of iron oxide (USPIO) enhanced imaging
Vascular reactivity	Hemodynamic response imaging (HRI)
Maintenance of corticomedullary sodium gradient	^23^Na-MRI

*(Patho)physiological processes in the kidney that can be assessed with functional MRI to date. Although a lower corticomedullary sodium gradient has been demonstrated in kidney allografts in comparison to native kidneys, sodium imaging has neither been able to reflect kidney function in kidney transplantation patients (Moon et al., 2014), nor in healthy individuals with a variety of eGFRs (Haneder et al., 2013), and is therefore not discussed in detail. However, it must be pointed out that preliminary experimental results in other kidney disease states, such as acute tubular necrosis, have shown changes in sodium imaging (Maril et al., 2006; Atthe et al., 2009), Similar to sodium MRI, hemodynamic response imaging has only been applied in experimental settings in animals thus far (Milman et al., 2013, 2014)*.

**Figure 1:**
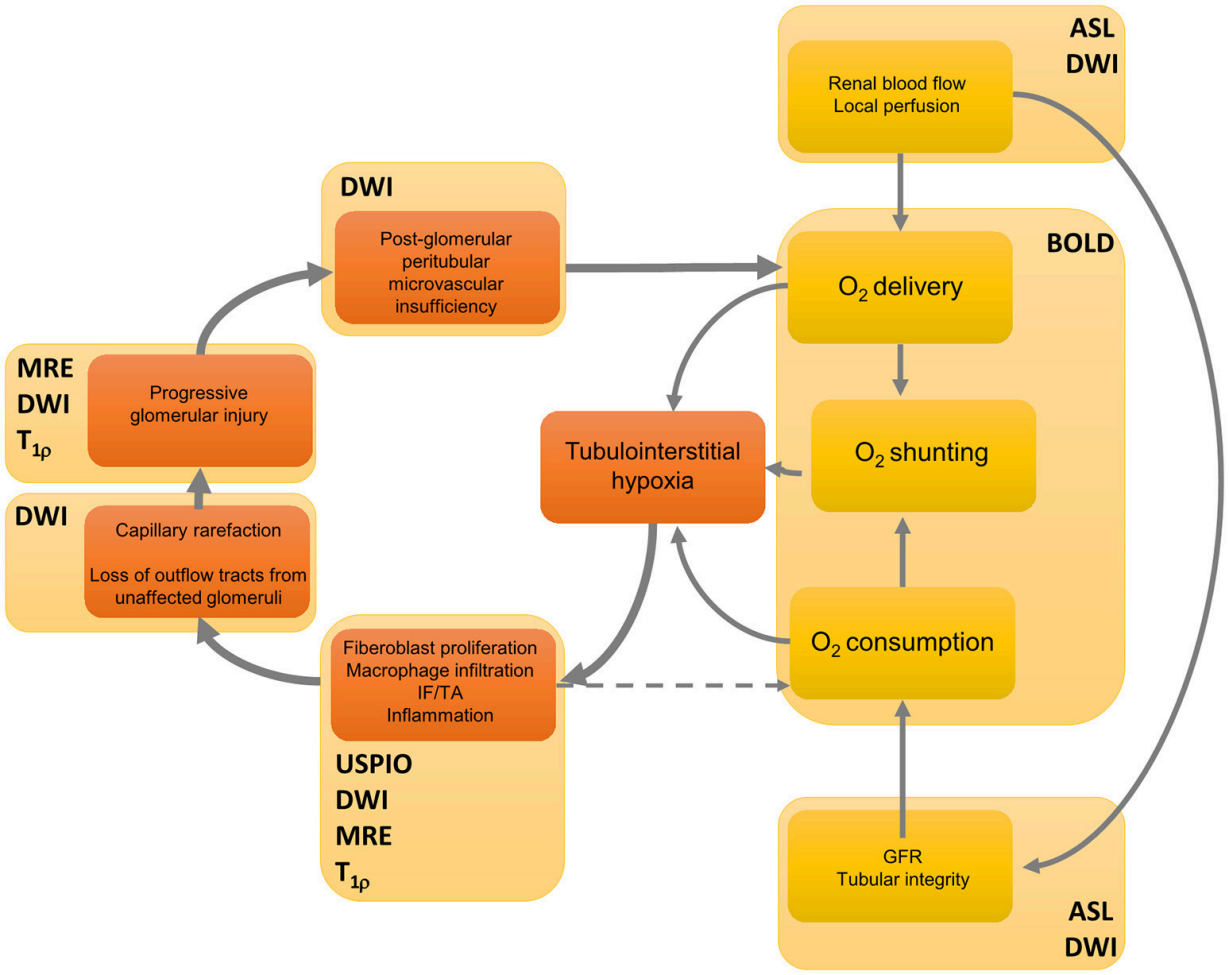
**A feedforward loop in the hypothesized processes of the development of fibrosis in chronic kidney disease, and their non-invasive modes of detection**. Renal blood flow determines delivery of oxygen, but also the glomerular filtration rate and thus oxygen consumption. Moreover, arteriovenous shunting has an effect on total oxygen balance. The initial event that gives rise to kidney hypoxia and thus to scarring could have a specific etiology, yet increased oxygen consumption is also caused by a variety of factors such as mitochondrial uncoupling following reperfusion immediately after transplantation (Evans et al., 2015). Interstitial hypoxia results in active inflammation and fibroblast proliferation directly mediated through hypoxia by cytokines such as transforming growth factor β1, which leads to interstitial fibrosis and tubular atrophy (IF/TA) (Fine et al., 2000). Active inflammation also contributes to hypoxia since it consumes energy. Then fibrotic foci infiltrate neighboring structures, causing further damage to previously unaffected tissue, which eventually has a negative effect on oxygen supply. For several links in the chain of events MRI techniques are displayed that yield visual information at a given functional event. Although many steps of the process are likely sensitive to one or another technique, no technique is specific for a single event. Furthermore, there is currently no imaging technique that can directly display tubulo-interstitial hypoxia. See Table 1 for denotation of abbreviations. DWI also encompasses DTI. This figure was modified from Fine and Norman (2008) (Elsevier) and Evans et al. (2013) (John Wiley and Sons) with permission from the publishers.

The essence of the chronic hypoxia theory is shown in Figure 1, along with the MRI techniques that are proposed to unravel specific links in the pathophysiologic chain of CAN. Based on this theory, some (functional) techniques deserve special interest for their potential in long-term follow-up: sequences that visualize (1) kidney perfusion and oxygenation, (2) parenchymal inflammation, and (3) kidney scarring. These techniques will be discussed in more detail later (Section Clinical experience with functional MRI in kidney allografts).

Thus far, the focus of interest has been on non-invasive diagnosis of the cause of acute allograft dysfunction. In this setting, non-functional MRI techniques possess unique qualities to assess morphologic abnormalities such as urinary obstruction (Kalb et al., 2008) and therefore better suit this application. Given the mechanisms underlying chronic kidney allograft dysfunction, we expect that the biggest gains with functional MRI can possibly be made in long-term follow-up.

In recent years, several existing and new gadolinium-free functional MRI techniques that measure various aspects of kidney function have advanced to the stage that they can be used in clinical practice. Below we discuss these in more detail.

## Clinical experience with functional MRI in kidney allografts

### Blood oxygen level dependent (BOLD) MRI

**Figure 2:**
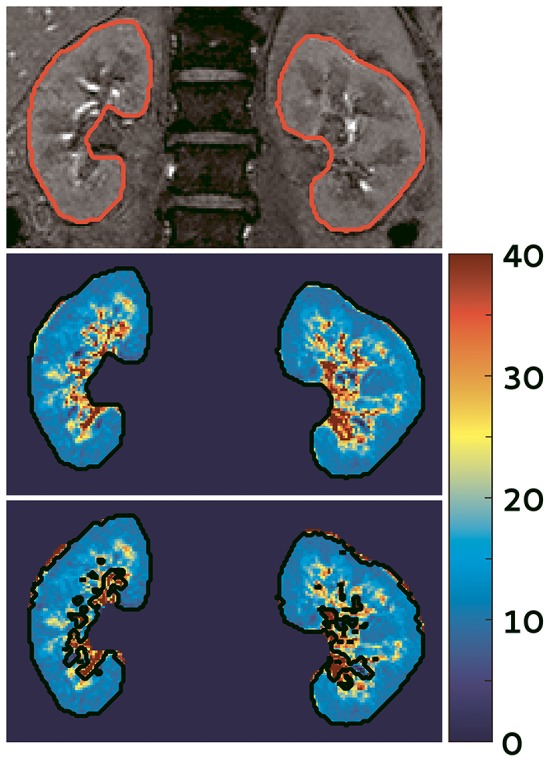
**BOLD image of the kidney acquired at our center in a hypertensive subject (unpublished data). Top:** one of the images used to calculate the ${\text{R}}_{2}^{*}$ maps; **middle:** ${\text{R}}_{2}^{*}$ map of the kidneys, note the difference between the well-oxygenated renal cortex (outer blue part) and the relatively hypoxic medulla (inner green/yellow parts). The scale denotes ${\text{R}}_{2}^{*}$ values in s^−1^, a low ${\text{R}}_{2}^{*}$ indicates higher oxygenation; **bottom:** automatic filter applied to exclude the renal vessels and urine collecting system.

BOLD MRI is used to obtain a measure for oxygenation (Figure 2). For a background of this imaging technique we refer to Pruijm et al. (2016) who have recently described renal BOLD MRI and its inherent limitations in detail in Frontiers in Physiology. BOLD MRI should be interpreted carefully, as also stated by Pruijm et al. (2016). Of special importance is the oxygen-hemoglobin dissociation curve. Since oxyHb releases oxygen more easily at lower oxygen levels, the amount of deoxyHb does not have a linear relationship with blood oxygenation (O'Connor et al., 2006; Leong et al., 2007). In the kidneys, BOLD MRI is therefore less sensitive to oxygenation changes in the highly oxygenated cortex (Prasad, 2006). Lastly, renal oxygenation can change due to increased consumption of oxygen by active processes in the kidney or decreased perfusion (Heyman et al., 2008). Strategies are now sought after that enable deduction of tissue *p*O_2_s from ${\text{R}}_{2}^{*}$ values using mathematical models, e.g., the two-step model by Zhang et al. (2014b) Interestingly, hemodynamic changes should not only be considered as a confounder in interpretation of BOLD images. In hemodynamic response imaging (HRI) air composition is intentionally modified (hypercapnia, hypercapnia plus hyperoxia) while at the same time multiple BOLD images are acquired. In this way hemodynamic changes due to Bohr-effect and CO_2_-induced vasodilation are provoked, for instance in the kidney, which enables evaluation of regional vascular reactivity. To date, only two studies in experimental animals have investigated kidney HRI (Milman et al., 2013, 2014).

Since the introduction of BOLD MRI in nephrology various researchers have investigated this technique in kidney transplant recipients (Sadowski et al., 2005, 2010; Djamali et al., 2006, 2007; Thoeny et al., 2006; Han et al., 2008; Park et al., 2012, 2014; Xiao et al., 2012; Liu et al., 2014). Table 2 lists studies in which BOLD MRI was used to assess malfunctioning kidney allografts. There is ample evidence that medullary ${\text{R}}_{2}^{*}$ values decrease in allografts with acute impaired function, possibly indicating regional blood *hyperoxia*. However, as explained above, changes in perfusion and hemoglobin content (blood volume, hematocrit) can also explain the decrease in ${\text{R}}_{2}^{*}$. In one study medullary and cortical blood hyperoxia as measured with BOLD MRI were related to respectively medullary and cortical hypoperfusion (Sadowski et al., 2010) suggesting that decreased oxygen consumption is the main reason for the hyperoxia observed in kidney allograft dysfunction. The most probable explanation for this would be that the GFR is reduced due to hypoperfusion, with consequently reduced solute delivery to the nephron and thus reduced regional oxygen consumption. Observation of improved medullary oxygenation during controlled hypotension demonstrated by Brezis et al. supports this explanation (Brezis et al., 1994).

**Table 2:** **Blood Oxygen Level Dependent (BOLD) MRI**.

**Study**	**Control subjects**	**Patients**	**Distinction between AR and ATN?**	**Field strength (T)**	M${\text{R}}_{2}^{*}$	C${\text{R}}_{2}^{*}$
Sadowski et al., 2005	NG	AR	*P* < 0.01	1.5	↓	NS
		ATN			NS	↑
Djamali et al., 2006	NG	AR	*P* < 0.05	1.5	↓	NS
		ATN			↓	NS
Thoeny et al., 2006	HV	NG	N/A	1.5	↓	NS
Djamali et al., 2007	HV	CAN	N/A	1.5	↓	↓
Han et al., 2008	NG	AR	*P* < 0.01	1.5	↓	↓
		ATN			↑	↑
Sadowski et al., 2010	NG	AR	*P* < 0.05	1.5	↓	NS
		ATN			NS	NS
Park et al., 2012	HV	AR	N/A	3.0	↓	NS
		NG			=	=
Xiao et al., 2012	HV	AR	N/A	1.5	↓	↓
		NG			=	=
Liu et al., 2014	NG	AR	*P* < 0.01	3.0	↓	NS
		ATN			NS	NS
Park et al., 2014	NG	AR	NS	3.0	↓	↓
		ATN			↓	↓

Medullary (M${\text{R}}_{\text{2}}^{*}$) and cortical (C${\text{R}}_{\text{2}}^{*}$) ${\text{R}}_{\text{2}}^{*}$ values were determined in malfunctioning kidney allografts. Values were compared to control subjects: either transplant patients with normal graft function (NG), or healthy volunteers (HV). AR denotes acute rejection, ATN acute tubular necrosis, CAN chronic allograft nephropathy, NS no significant difference and N/A not applicable.

Moreover, significant differences in medullary ${\text{R}}_{2}^{*}$ (M${\text{R}}_{2}^{*}$) values between acute rejection and acute tubular necrosis have been reported with BOLD MRI (Table 2). Differences in cortical ${\text{R}}_{2}^{*}$ (C${\text{R}}_{2}^{*}$) values between native kidneys or allografts with normal function and allografts with impaired function are often not significant, which is remarkable given that sodium is mainly reabsorbed in the cortex. It would therefore be expected that this is reflected by BOLD parameters, since the cortex accounts for the major part of total oxygen consumption, as it receives most of total renal blood flow (Brezis et al., 1984). Possibly this is explained by the lower sensitivity of BOLD MRI to changes in the renal cortex, as explained above. However, signal intensity due to BOLD effects increases almost linearly to main field strength up to 7T (Seehafer et al., 2010). Hence, with current technology, it is conceivable that differences in C${\text{R}}_{2}^{*}$s are invisible due to low signal-to-noise ratios. Advances in experimental settings in ultrahigh field scanners may establish better resolution of cortical pathophysiology.

As mentioned, experience in the chronic phase post-transplantation is very limited. Djamali et al. investigated ${\text{R}}_{2}^{*}$ parameters in 10 transplantation patients clinically diagnosed with CAN that were at least 12 months post-transplantation, and found decreased values for C${\text{R}}_{2}^{*}$ and M${\text{R}}_{2}^{*}$ compared to healthy volunteers (Djamali et al., 2007). However, one of the inclusion criteria in this study was that CAN patients suffered at least KDOQI stage 3, which implies that fibrosis was probably abundantly present in all allografts studied. It would be presumptive therefore to draw any conclusions from this study with regard to the role of hypoxia in development of CAN. Interestingly though, markers of oxidative stress in serum and urine correlated significantly with ${\text{R}}_{2}^{*}$ values in CAN patients. Furthermore, M${\text{R}}_{2}^{*}$ was studied repeatedly in 15 transplanted kidneys. As compared with baseline readings, obtained before renal harvesting, M${\text{R}}_{2}^{*}$ declined by 8.3% (*P* = 0.06) over 2 years follow-up (Niles et al., 2016). Since allograft GFR in these patients even improved during follow-up by 33.3% (*P* < 0.01) compared to corrected baseline GFR, these results suggest a role for disturbed oxygen balance in development of subclinical chronic damage. Continuous follow-up of these patients beyond 2 years would be interesting to further test this hypothesis. In contrast, results with BOLD MRI in CKD are inconsistent with the above, since most studies demonstrate no difference in C${\text{R}}_{2}^{*}$ and M${\text{R}}_{2}^{*}$ among CKD patients with different characteristics, such as disease stage (Neugarten and Golestaneh, 2014). However, this does not imply that the chronic hypoxia theory should be rejected, since incompletely understood oxygen balance and other reasons inherent to BOLD MRI already mentioned may confound interpretation of these results (Neugarten and Golestaneh, 2014).

It follows from these studies that the relationship between BOLD parameters and kidney function in kidney allografts is not fully understood. Confusingly, decreased values for ${\text{R}}_{2}^{*}$ are found in malfunctioning kidney allografts (which contain at least small fibrotic foci), whereas increased ${\text{R}}_{2}^{*}$s corresponding to blood hypoxia are found in other kidney diseases characterized by fibrosis (Neugarten and Golestaneh, 2014). For better interpretation of changes in ${\text{R}}_{2}^{*}$ values, it would be interesting to combine BOLD MRI with perfusion. Thus, on the one hand clinical translation of BOLD MRI remains difficult, but on the other hand it is arguable that BOLD parameters should be used if they are prognostic of allograft dysfunction, despite our lack of understanding of underlying pathophysiology.

### Arterial spin labeling (ASL)

**Figure 3:**
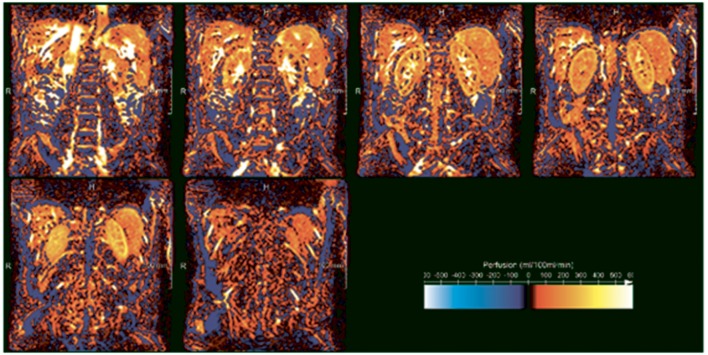
**ASL perfusion images of the kidney in a healthy volunteer acquired at our center (unpublished data)**. White and yellow denote high perfusion, while orange and red denote low perfusion. The difference between the highly-perfused cortex and less highly perfused medulla is clearly visible. In the three upper panels, starting on the left, the high signal intensity regions in the kidney centers reflect the pyelocalceal system.

Arterial Spin Labeling (ASL) is used for mapping of arterial blood flow and cortical perfusion at capillary level. In ASL, spins are labeled in the arterial phase (Ferré et al., 2013). Following a delay time to allow the labeled spins to flow in to the capillaries and diffuse into the tissue, an MRI of the tissue is acquired. The spins lose some of the magnetization due to T_1_ decay during the delay time. Combining this with a similar image acquired without applying the label, allows for renal cortical perfusion to be assessed (Ferré et al., 2013) which results in ASL maps (Figure 3). In this way rate of cortical diffusion and thus degree of cortical perfusion can be assessed. In fact, ASL is an alternative to DCE-MRI, with the difference that for ASL moving blood is used as an endogenous CA, instead of a GBCA. A moderate correlation (*r* = 0.66) between the two techniques was found in a small study in which total kidney blood flow was determined in 19 healthy volunteers (Wu et al., 2011). Although substantial differences between DCE and ASL parameters are present in all studies in which the two techniques are compared, both techniques possess the ability to detect impaired kidney perfusion (Zimmer et al., 2013). ASL parameters are highly reproducible in healthy subjects, (Cutajar et al., 2012, 2014; Gillis et al., 2014; Kistner et al., 2015) with better reproducibility than DCE-MRI (Cutajar et al., 2014).

Three studies in kidney transplantation patients have demonstrated a good correlation between kidney function and cortical perfusion as assessed with ASL (Table 3) (Lanzman et al., 2010; Heusch et al., 2013, 2014) These studies also included patients with stable eGFR in the chronic phase (> 1 year) post-transplantation, but given its cross-sectional design yielded no information on the course of kidney perfusion in patients with stable allograft function. However, the study by Niles et al. (2016) demonstrated that after 2 years follow-up, in eight kidney allografts with stable function, cortical perfusion decreased by 34.2% (*P* < 0.001) compared to baseline. According to Figure 1, reduced cortical perfusion can lead to a decrease in GFR and over time to cortical hypoxia due to decreased oxygen delivery, which may initiate the “hypoxia loop.” Besides, another subset of participants that was given losartan, starting 3 months post-transplantation, achieved considerably better cortical perfusion upon termination of follow-up (22.9% more in comparison to the participants without losartan, *P* < 0.05).

**Table 3:** **Arterial Spin Labeling (ASL)**.

**Study**	**Control subjects**	**Patients**	**Field strength (T)**	**Cortical perfusion**
Lanzman et al., 2010	NG, HV	IG (>20% increase in SCr)	1.5	↓*P* < 0.001
Heusch et al., 2013	N/A	Kidney allograft recipients with range of eGFRs	1.5	↓*P* < 0.05
Heusch et al., 2014	NG	IG (eGFR ≤ 30 mL/min/1.73 m^2^)	1.5 and 3.0	↓*P* < 0.0001

*Arterial Spin Labeling (ASL) in kidney allografts. Impaired graft function (IG) was defined differently in all three studies, and patients were compared to transplant patients with normal graft function (NG), or healthy volunteers (HV). In the study by Heusch et al. (2013) a set of ASL maps was correlated to the corresponding values for eGFR (r = 0.63). N/A denotes not applicable*.

Preliminary results demonstrated the possibility to assess single-kidney GFR with ASL (He et al., 2014) which is an advantage over eGFR based on SCr. This might be of use in the transplantation work-up of living donors and donors after brain death to determine whether one of both kidneys would be preferred over the other for harvesting based on difference in kidney function. A comparison between cortical perfusion and histopathology has not been made in human kidney allograft recipients to date. However, the percentage of affected tubules in kidney biopsy in mice with acute kidney injury was shown to correlate (*r* = 0.73) with relative kidney perfusion as determined with ASL (Hueper et al., 2014).

### Diffusion weighted imaging (DWI)

Diffusion weighted imaging (DWI) measures the diffusion of water molecules in tissue. Since water motion in tissue is restricted, its diffusion constant differs from that of free water and is therefore described with the apparent diffusion coefficient (ADC). Applying a diffusion weighting (indicated by the b-value) causes loss of signal which is proportional to the ADC and can be modeled using a mono-exponential function. Seven studies investigated mono-exponential DWI in kidney transplantation patients (Table 4) (Thoeny et al., 2006; Blondin et al., 2011; Hueper et al., 2011, 2016; Lanzman et al., 2013; Park et al., 2014; Fan et al., 2016) Next to the microscopic diffusion of water the signal measured in DWI is also sensitive to the microcirculation. As such, the measured ADC using low b-values reflects both diffusion and perfusion and the signal can be better described using a bi-exponential model that accounts for contribution of both compartments, which is also known as Intravoxel incoherent motion (IVIM) imaging. Vermathen et al. investigated the variability in eight transplantation patients with stable kidney function using the bi-exponential model (Vermathen et al., 2012). After two sessions of DWI (7 ± 3 months and 32 ± 2 months after transplantation) coefficients of variation within and between individuals were low: <3.5 and <5.9%, respectively (Vermathen et al., 2012). A second and more important argument in favor of the use of the bi-exponential model is that contributions of pseudodiffusion and structural diffusion to total ADC can be assessed separately, and thus IVIM parameters may reveal structural tubular damage (Notohamiprodjo et al., 2015).

**Table 4:** **Diffusion Weighted Imaging (DWI)**.

**Study**	**Control subjects**	**Patients**	**Field strength (T)**	**Cortical ADC**	**Medullary ADC**
Thoeny et al., 2006	HV	NG	1.5	↓^*^	↓^*^
Blondin et al., 2011	NG	IG	1.5	↓^*^	N/A
Hueper et al., 2011	HV	IG	1.5	↓^*^	↓^*^
Lanzman et al., 2013	G-MG	IG	3.0	↓^*^	↓^‡^
Park et al., 2014	NG	IG	3.0	↓^*^	↓^†^
Fan et al., 2016	HV	NG IG	3.0	NS ↓°	↑^*^↓°
Hueper et al., 2016	NG	DG	1.5	↓^*^	↓^*^

*Several studies found decreased ADC values in patients with impaired allograft function (IG) as compared to patients with normal allograft function (NG), good to moderate allograft function (G-MG), and healthy volunteers (HV). N/A denotes not applicable, NS not significant*.

* *P < 0.01*,

† *P < 0.016*,

‡ *P = 0.01*,

° *No P-values were given for IG as compared to HV in this study*.

Another way of describing the signal measured with DWI is the tensor model, also known as Diffusion tensor imaging (DTI). Diffusion of water within a compartment such as a blood vessel or tubule is restricted to a specific direction by boundaries of the compartment. This phenomenon is known as anisotropy. If DWI is performed in at least six unique directions it becomes possible to describe the anisotropic diffusion using the diffusion tensor from which a quantitative measure of anisotropy can be derived, e.g., the fractional anisotropy (FA). Data can be displayed more sophisticatedly by showing intervoxal connectivity, which is called tractography. In tractography in each voxel the primary diffusion direction is identified and described by a vector (more precisely the direction of its primary eigenvalue, i.e., one of three eigenvalues that are determined in the calculation of the diffusion tensor) (Mukherjee et al., 2008). Interpolation of this vector field visualizes the diffusion direction as fiber tracts (Mukherjee et al., 2008). In this way, DTI might enable the assessment of tubular and vascular membrane integrity (McRobbie et al., 2006).

**Figure 4:**
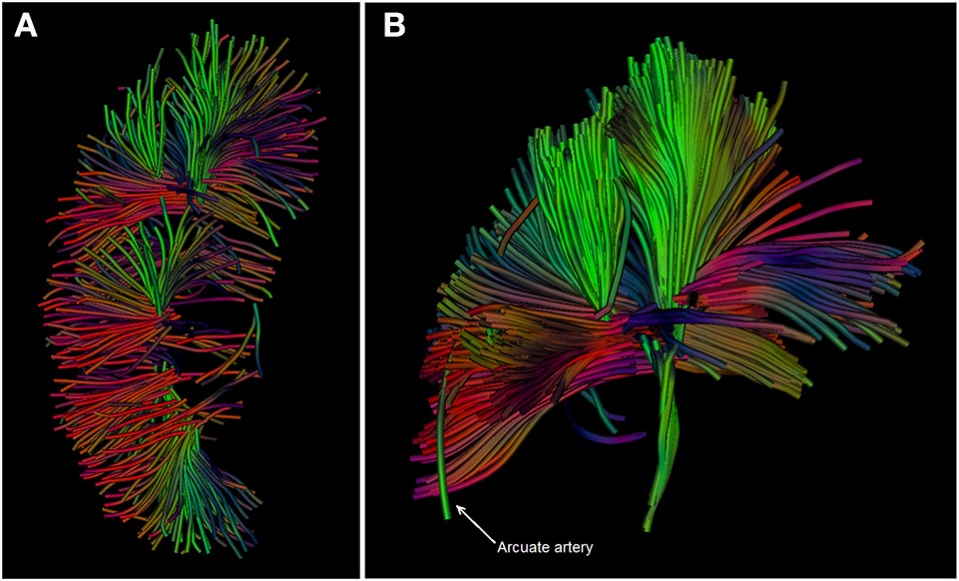
**(A,B):** DTI images of the kidney in a healthy volunteer acquired at our center. Diffusion tracts running from the cortex through the medullary pyramids are depicted. The different colors indicate the net direction of diffusion. Source data for these images were retrieved from an existing dataset in our center (van Baalen et al., 2016).

Fan et al. used DTI to study kidney allograft function early after transplantation and found significant differences between medullary and cortical FA in all subjects: healthy volunteers, and allograft recipients with good, moderate, and severely impaired function (Fan et al., 2016). Medullary FA was larger than cortical FA, probably reflecting the highly organized radial structure of the tubular system, clearly visible in Figure 4, in contrast to the mesh of small vessels and glomeruli found in the cortex (Fan et al., 2016). This element in particular relates to the clinical use of DTI, since only medullary FA correlates with eGFR (Hueper et al., 2011, 2016; Lanzman et al., 2013). Similar to other studies mentioned above, lower cortical and medullary ADCs were found in patients with impaired graft function (Hueper et al., 2011; Lanzman et al., 2013; Fan et al., 2016). In addition, Cheung et al. related a decline in medullary FA and ADC to histologic injury (medullary cast formation and cell necrosis) seen in ischemic reperfusion injured kidneys of mice (Cheung et al., 2010). Hueper et al. also reported correlations between diffusion parameters and injury (*r* = −0.63 in FA and *r* = −0.65 in ADC_mono_) (Hueper et al., 2016). Although ADC coefficients show correlation with eGFR and are discriminative between normal and impaired allograft function in the acute phase, chronically malfunctioning allografts have only been studied using DWI in a small number of subjects. Therefore, whether DWI is applicable for long-term follow-up is as yet unknown.

### T_1_ in the rotating frame (T_1ρ_)

A relatively new technique in kidney imaging is T_1_ in the rotating frame (T_1ρ_) (He et al., 2011). This modality is highly sensitive to interactions of large macromolecules including collagen (fibrosis) with water molecules. T_1ρ_ is a well-established imaging technique in orthopedic radiology, originally being designed for measuring cartilage in skeletal joints (Li et al., 2011). A recent study applied this modality to the kidney (Rapacchi et al., 2015). In this study, that also investigated ASL, BOLD MRI, and DWI, a receiver operating characteristic analysis was conducted for medullary T_1ρ_ relaxation time which yielded an area under the curve of 94% (95% CI: 82–100%) for prediction of lupus nephritis (Rapacchi et al., 2015). Since fibrosis is abundantly present in kidney allografts with impaired function, and, given these initial results that T_1ρ_ appears to allow accurate visualization of fibrosis in kidney tissue, this technique is worth investigation in kidney allografts.

### Ultrasmall superparamagnetic particles of iron oxide (USPIO) enhanced imaging

Labeling of macrophages is possible with infusion of ultrasmall superparamagnetic particles of iron oxide (USPIO), since these particles diffuse into extravascular space quickly after infusion and are then taken up by macrophages (Gellissen et al., 1999). Similar to deoxyHb in BOLD MRI, these particles influence the main magnetic field. Therefore, the discrepancy on ${\text{T}}_{2}^{*}$ weighted images before and after infusion of ferumoxytol (i.e., USPIO) relates to regional macrophage infiltration, e.g., in glomeruli. The uptake of USPIO by macrophages in a rat model of acutely rejected kidney allografts was demonstrated more than a decade ago (Zhang et al., 2000; Ye et al., 2002) but Beckmann et al. were the first to show macrophage infiltration in rats with chronic allograft inflammation by USPIO enhanced imaging (Beckmann et al., 2003).

The clinical relevance of glomerular macrophage count (GMC) in kidney biopsies was demonstrated by Sentís et al., who found that the GMC is predictive of death-censored graft failure up to 500 days after biopsy (Sentís et al., 2015). The average time between transplantation and performance of kidney biopsy was 18 days, and therefore no conclusions could be drawn on the relevance of macrophages in chronic inflammation (Sentís et al., 2015). However, it suggests that the GMC may render valuable information in the chronic phase as macrophages play an important role in chronic inflammation leading to interstitial fibrosis and tubular atrophy (Dang et al., 2012). In a small study conducted in 12 patients, including five kidney transplantation patients, signal variation on ${\text{T}}_{2}^{*}$ images correlated (*r* = −0.7, *P* = 0.011) to cortical macrophage infiltration (Hauger et al., 2007). A drop in medullar signal intensity could also be seen in three patients with acute tubular necrosis, which typically manifests in the renal medulla and is therefore not accompanied by cortical macrophage infiltration (Hauger et al., 2007). Apart from its potential as indicator of macrophage infiltration in functional imaging, USPIO have been suggested as a safer alternative to gadolinium in patients with severely impaired kidney function (Bashir et al., 2015; Mukundan et al., 2016). Still, it is of note that ferumoxytol, which in daily practice is mostly used for the management of iron-deficiency anemia in chronic kidney disease patients, is sporadically associated with hypersensitivity which could ultimately result in anaphylaxis. An incidence rate of 34.1 (95% CI: 23.1–50.0) per 100,000 new users for anaphylaxis was reported (Wang et al., 2015) but it must be pointed out that this study reported lower rates of anaphylaxis than clinical trials, possibly because cases of minor hypersensitivity reactions are not routinely registered if not in the setting of a clinical trial (Wang et al., 2016). It is also still unknown if rapid ferumoxytol infusion leads to increased incidence of anaphylaxis compared to slow infusion (Wang et al., 2015) but if used as a marker of macrophage infiltration slow infusion of ferumoxytol would suffice. In conclusion, experience with USPIO is limited to small studies. Large clinical trials are needed to prove safety of USPIO enhanced imaging (Finn et al., 2016).

### Magnetic resonance elastography (MRE)

Research in healthy volunteers has shown that magnetic resonance elastography (MRE) is feasible and reliable for the assessment of kidney stiffness (Rouvière et al., 2011; Low et al., 2015). In an animal model of renal artery stenosis MRE could detect medullary stiffness, which reflected medullary fibrosis (Korsmo et al., 2013). The potential to acquire 3D images with MRE is a great advantage over ultrasound (US), which only yields 2D images, although spatial resolution of MR imaging is inferior to US (Grenier et al., 2013). Human research in the field of MRE is limited, because US is far more practical, less expensive and less labor intensive in the clinical setting than MRE.

## Summarizing discussion and conclusion

Novel imaging modalities have to pass the standard threshold of development before they are clinically implemented (Sado et al., 2011). Some of the MRI techniques discussed here are now at the stage where their disease-predictive nature has been demonstrated. All the above techniques correlate with functional parameters, mostly eGFR, and sometimes also with histopathology. However, these correlations are relatively modest, due to limitations in current interpretation software-tools, but also to the inherent insensitivity of eGFR to limited nephron loss (Pascual et al., 2012).

Most functional imaging techniques are not routinely used in clinical practice. However, chances are high that eventually imaging techniques, alone or in combination, will yield data precise enough to accurately diagnose (sub)clinical kidney disease. In the future MRI could become feasible as a screening tool in long-term follow-up of kidney allografts. Longitudinal information on active inflammation and existing fibrosis, a combination that was identified as a strong predictor for allograft loss (Haas, 2014) could be gained, and in this way MRI could contribute to clinical decision making. For instance, MRI findings suggestive for subclinical damage could support the decision to proceed to kidney biopsy in the absence of clinical symptoms. Lastly, the whole kidney fibrosis percentage can be estimated, which will allow better appreciation of the extent of fibrosis than with a biopsy.

Admittedly, progress made in the past decade toward clinical implementation is not satisfying. This may be partially explained by the fear of nephrogenic systemic fibrosis due to GBCAs. It should therefore be stressed that with current technology administration of GBCAs has become unnecessary in many cases. Awareness of the potential of functional MRI in long-term follow-up of kidney transplantation patients among nephrologists is warranted (Zhang et al., 2014a). This review illustrates that functional MRI has the potential to probe several pathophysiological mechanisms involved in kidney allograft dysfunction, and is a promising tool in long-term follow-up of kidney transplantation patients.

## Author contributions

MvE participated in the initial literature search, data analysis, and interpretation, drafting of the manuscript, study group discussions, and approved the final version of the article; AvZ participated in the literature search, data analysis and interpretation, drafting of the manuscript, study group discussions, and approved the final version of the article; AdB and MF participated in data analysis and interpretation, drafting of the article, study group discussions, and approved the final version of the article; TN participated in the literature search, data analysis and interpretation, drafting of the manuscript, study group discussions, and approved the final version of the article; JJ participated in data analysis and interpretation, drafting of the article, and approved the final version of the article; TL participated in data analysis and interpretation, drafting of the article, attending study group discussions, study supervision and approved the final version of the article; MV participated in drafting of the article, study group discussions, study supervision and approved the final version of the article.

## Funding

This work was supported by the Dutch Kidney Foundation (Nierstichting Nederland) [grant number 150KK119]. The Dutch Kidney Foundation had no role in deciding on the study design, or in the collection, analysis, and interpretation of data; neither has this funder contributed to writing the article nor to making the decision to submit the article for publication.

### Conflict of interest statement

The authors declare that the research was conducted in the absence of any commercial or financial relationships that could be construed as a potential conflict of interest.

## Abbreviations

ADC: Apparent diffusion coefficient
AKI: Acute kidney injury
ASL: Arterial spin labeling
BOLD: Blood oxygen level dependent
CAN: Chronic allograft nephropathy
CKD: Chronic kidney disease
DCE: Dynamic contrast-enhanced
(de)oxyHb: (De) oxyhemoglobin
DTI: Diffusion tensor imaging
DWI: Diffusion weighted imaging
(e)GFR: (Estimated) glomerular filtration rate
FA: Fractional anisotropy
GBCA: Gadolinium-based contrast agent
GMC: Glomerular macrophage count
IVIM: Intravoxel incoherent motion
MRE: Magnetic resonance elastography
MRI: Magnetic resonance imaging
SCr: Serum creatinine concentration
US: Ultrasound
USPIO: Ultrasmall superparamagnetic particles of iron oxide.
